# Automatic segmentation of meningioma from non-contrasted brain MRI integrating fuzzy clustering and region growing

**DOI:** 10.1186/1472-6947-11-54

**Published:** 2011-08-26

**Authors:** Thomas M Hsieh, Yi-Min Liu, Chun-Chih Liao, Furen Xiao, I-Jen Chiang, Jau-Min Wong

**Affiliations:** 1 Institute of Biomedical Engineering, and College of Medicine, National Taiwan University, Taipei, Taiwan; 2 Division of Plastic Surgery, Department of Surgery, National Taiwan University Hospital, Taipei, Taiwan; 3 Division of Neurosurgery, Department of Surgery, National Taiwan University Hospital, Taipei, Taiwan

## Abstract

**Background:**

In recent years, magnetic resonance imaging (MRI) has become important in brain tumor diagnosis. Using this modality, physicians can locate specific pathologies by analyzing differences in tissue character presented in different types of MR images.

This paper uses an algorithm integrating fuzzy-c-mean (FCM) and region growing techniques for automated tumor image segmentation from patients with menigioma. Only non-contrasted T1 and T2 -weighted MR images are included in the analysis. The study's aims are to correctly locate tumors in the images, and to detect those situated in the midline position of the brain.

**Methods:**

The study used non-contrasted T1- and T2-weighted MR images from 29 patients with menigioma. After FCM clustering, 32 groups of images from each patient group were put through the region-growing procedure for pixels aggregation. Later, using knowledge-based information, the system selected tumor-containing images from these groups and merged them into one tumor image. An alternative semi-supervised method was added at this stage for comparison with the automatic method. Finally, the tumor image was optimized by a morphology operator. Results from automatic segmentation were compared to the "ground truth" (GT) on a pixel level. Overall data were then evaluated using a quantified system.

**Results:**

The quantified parameters, including the "percent match" (PM) and "correlation ratio" (CR), suggested a high match between GT and the present study's system, as well as a fair level of correspondence. The results were compatible with those from other related studies. The system successfully detected all of the tumors situated at the midline of brain.

Six cases failed in the automatic group. One also failed in the semi-supervised alternative. The remaining five cases presented noticeable edema inside the brain. In the 23 successful cases, the PM and CR values in the two groups were highly related.

**Conclusions:**

Results indicated that, even when using only two sets of non-contrasted MR images, the system is a reliable and efficient method of brain-tumor detection. With further development the system demonstrates high potential for practical clinical use.

## Background

In recent years, magnetic resonance imaging (MRI) has become an important modality for neurological image diagnosis. A noticeable body of research reported detection of pathology within the neurological system based on the differing tissue characters in T1-weighted and T2-weighted MR images [1,2]. In this area of study, the segmentation of brain tumor images represents important and challenging work [3]. Automatic algorithms developed to replace the time-consuming manual segmentation include level-set operation [4], support vector machine (SVM) [5,6], k-nearest neighbor (KNN) [7], watershed algorithm [8]. or the use of a brain atlas [9,10]. Among them, many previous studies applied the fuzzy clustering class of algorithms. Dou [11] used a framework based on "fuzzy information fusing" for brain tumor segmentation, Liu [12] used fuzzy connectedness to compute the brain tumor volume. Khotanlou [13] used "fuzzy-possiblistic c-mean (FPCM)" combined with the asymmetrical prior knowledge in 3D brain tumor segmentation; and, in his another work [14], adopted the deformable model together with fuzzy clustering.

Bezdek first proposed the fuzzy c-means (FCM) algorithm in 1981 [15]. Since then it has become a popular clustering method which divides data into different groups according to their degree of attribution. Data may partially belong to more than one group, represented by a fuzzy membership value of between 0 and 1. During image analysis, each pixel is classified according to their attributes: a membership value of 1 means that a pixel contains only one specific tissue class; whereas a membership value of 0 means that a pixel does not contain that tissue class. Since the unsupervised FCM does not require training data researchers have widely used this method in the segmentation of MR images [16,17]. Clark [18] used FCM with the knowledge-based (KB) procedure in brain tumor segmentation. Emblem [19] used knowledge-based fuzzy c-means (FCM) clustering on multiple classes of MR image for glioma detection. Wafa [20] used multi-featured FCM and evidence theory on multimodal MRI for brain tumor segmentation, and Fletcher-Heath [21] used two-stage FCM combined with KB procedure on non-contrasted MR images for tumor segmentation. These assessments made use of the contrasted-enhanced MR image or more than two types of images as study materials. This, therefore, included a risk of possible reaction to the contrast medium, and also increased the overall calculation load.

After applying FCM, a defuzzification stage is usually performed in order to convert the fuzzy memberships into a clear-cut set. According to previous research [22], and prior experience, during this stage, data or images are usually too noisy and tend to become multiple small and fragmented pieces, which are usually too small to be properly grouped. Previous literatures proposed including the region relationship into the procedure as a possible solution to this drawback [23-25]. This study, therefore, attempts to adopt the region growing algorithm into the procedure, in order to effectively eliminate the fragment portion within the post-clustering images, and to avoid further errors in subsequent procedures.

This research uses images of menigioma as study materials. Unlike other forms of brain tumor, menigioma is one of the few benign tumors found in this region, so precise tumor margin detection can be curtail in complete surgical resection. Another characteristic of this tumor is that there is a greater possibility for it to be situated at the brain midline [26,27]. This particular feature poses a challenge to tumor location, because in the post-clustering KB selection process, many researchers locate tumors based on the symmetry of brain anatomy [28,29]. If the tumor is located in the midline, it cannot, therefore, be effectively detected at this stage.

This study used only non-contrasted T1 and T2-weighted images to develop a method based on the fuzzy-c-mean, together with the region-growing algorithm, to extract the meningioma from the MR image. The overall aim was to successfully complete tumor image segmentation, and at the same time, to effectively detect the midline tumor.

## Methods

### Data

MR images of 29 patients with meningioma were retrieved from the medical imaging department via the picture archiving and communication system (PACS) (This research had been approved by *National Taiwan University Hospital (NTUH) Research Ethics Committee (REC)*, Case No.: *201106064RC*).

All MR imaging was performed with 1.5 Telsa units (Signa HDx; GE Medical Systems, Milwaukee, Wisconsin, US) using an 8-channel phased array head coil. The MR protocol included:

1. Spin-echo T1-weighted imaging, with the following sequences: TR/TE = 500-700/20 ms, slice thickness 5 mm, gap 1.5 mm, number of average 1, matrix 512 × 512. The contrast-enhanced images (CET1) were also obtained using the same protocol after the contrast agent was injected.

2. Fast spin-echo T2-weighted imaging, with the following sequences: TR = 5000-6000/80-100 ms, echo-train length 18-24, slice thickness 5 mm, gap 1.5 mm, number of average 2, matrix 512 × 512.

All patients were examined by the same machine, with same sequence parameters. The images were obtained under well-controlled conditions by an experienced technician, to ensure geometrically-aligned orientations. An expert radiologist reviewed the whole series of images, and confirmed that there was no intensity inhomogeneity in the images, and consequently no need for intensity normalization.

**Figure 1:**
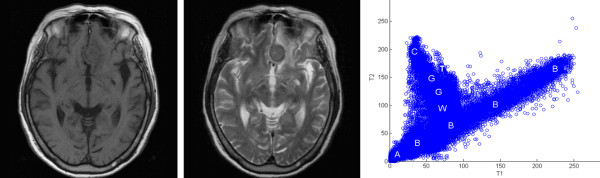
**Study material**. One of the 29 study groups of the MR images of menigioma is shown here, T1-wighted image (left), T2-weighted image (middle) and two-dimensional intensity histogram based on these 2 images (right).

The T1 and T2-weighted non-contrasted axial MR images were analyzed during the study. At the same time, the contrast-enhanced T1-weighted axial images (CET1) were used for manual tumor segmentation and the resulting "ground truth" (GT) was used to validate results, as shown in Figure 1.

### Pre-processing

**Figure 2:**
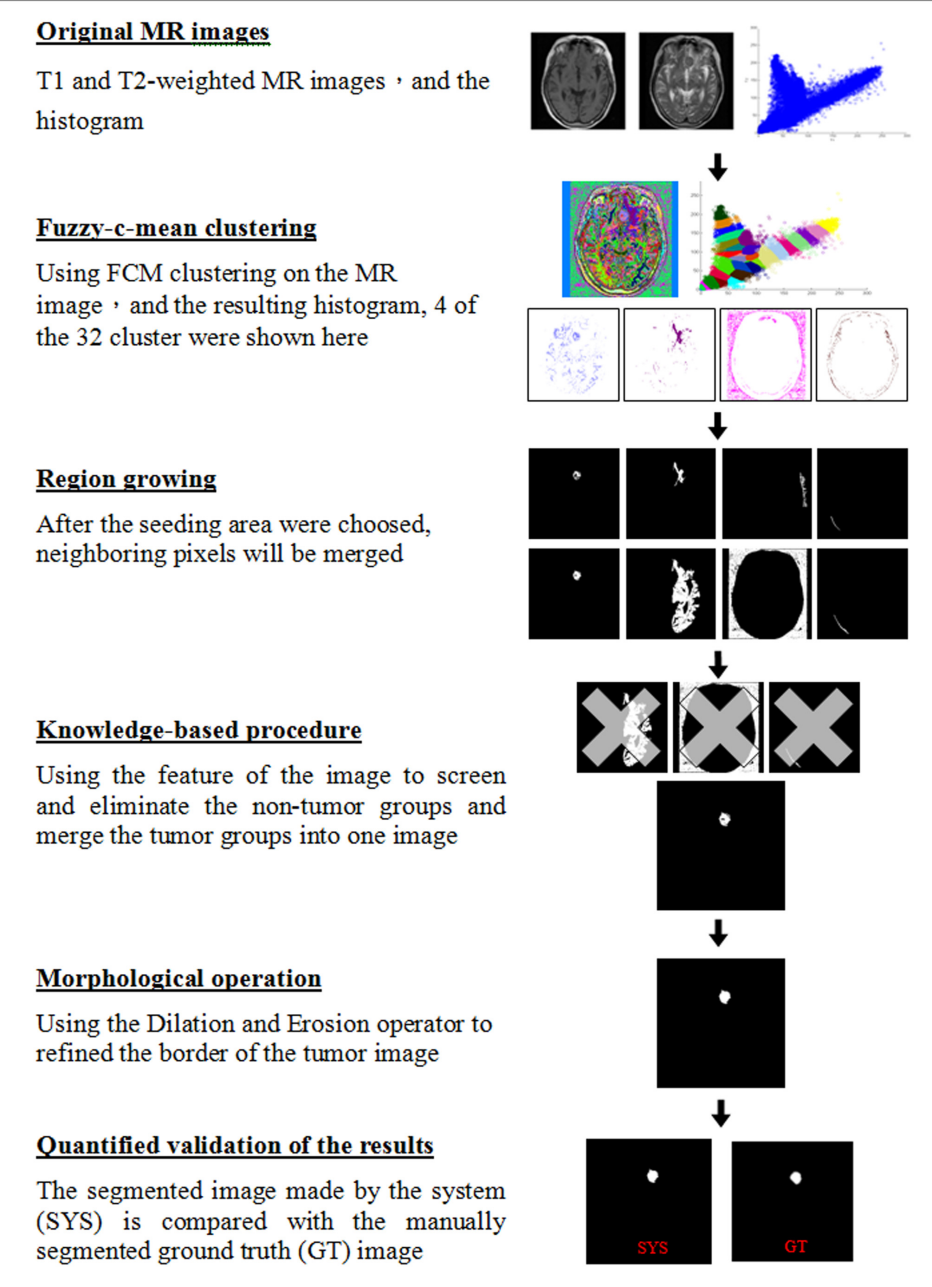
**Flow Chart of the research procedures**.

Before the clustering process, all images went through a co-registration procedure for quality control. Co-registration was performed using the Insight Toolkit (ITK) [30]. Affine transformation and mutual information were used formetric and linear interpolation during the registration process. A board certified neurosurgeon confirmed the results of registration before commencement of the tumor image extraction process. This is summarized in Figure 2.

### Fuzzy-c-mean clustering

Image segmentation was processed using a software package (Matlab 7.6, MathWorks, Natick, MA, USA). Based on the differences in gray levels of T1- and T2- weighted images, a two-dimensional intensity histogram, as shown in Figure 1, was created to represent the distribution of intensities in T1 and T2 images. For a pair of 8-bit images, the histogram consisted of a set of 256 × 256 bins, which counted the number of pixels falling in the given area of the (T1 intensity, T2 intensity) plane. A previously published FCM cluster algorithm was used to partition the two-dimensional histogram. The basic procedures can be briefly summarized as follows:

Let × = {X_1_, ..., X_n_} denote a pair of images with n pixels, and let X_k _= (X_k,T1_, X_k,T2_) be ordered pair of T1 and T2 intensities of the k-th pixel of X, 1 ≤ k ≤ n.

$$x_{k}=\left(x_{k,T1},x_{k,T2}\right)\in {ℜ}^{2},\forall x_{k}\in X$$

Step 1. Divide the dataset × = {X_1_, ..., X_n_} to c clusters

Set membership value u_ik _randomly, with the constraint:

$$u_{k}^{i}\in \left[0,1\right],\forall i,k;\sum_{i=1}^{c}u_{ik}=1,\forall k;$$

Step 2. Calculate the computer cluster center

$$J_{i}^{iter}=\frac{\sum_{k=1}^{n}{\left(u_{ik}^{iter}\right)}^{m}x_{k}}{\sum_{k=1}^{n}{\left(u_{ik}^{iter}\right)}^{m}},1\leq i\leq c,$$

where *m *> 1 is the exponential weight that regulates the influence of membership grades and (*t*) is the iteration number.

Step 3. Calculate objective function

$$J_{i}^{iter}=\sum_{k=1}^{n}{\sum_{i=1}^{c}\left(u_{ik}^{iter}\right)}^{m}{∥x_{k}-v_{i}^{iter}∥}^{2}$$

Step 4. Minimize objective function

*If |J^(iter) ^- J^(iter+1) ^| ≤ ϵ, end; *or else *t = t+1 *and return to step 2.

**Figure 3:**
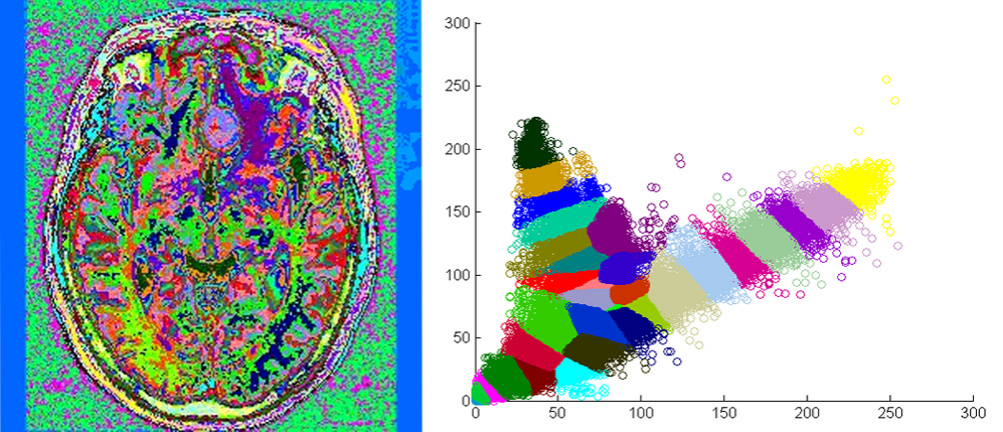
**Result after FCM clustering**. FCM clustering on MR images of the brain (left), and the histogram after defuzzification (right), a total of 32 groups of binary image were produced according to the colors zones shown on the histogram, each color represent a particular tissue character.

After FCM clustering, the histogram of the MR brain image resembled Figure 3. The concept of over-segmentation was adopted for better results, and data was initially defuzzified into 32 groups. By merging or eliminating these groups via the following steps, the number of groups decreased.

### Region growing

After FCM clustering, the image was divided into 32 binary images. Some of the images were too fragmented for proper grouping so the region-growing algorithm was adopted to solve this problem. This algorithm postulates that pixels in the same region have similar features. By selecting initial seed points and utilizing the similarity of seed and adjacent pixels, partitions are merged into larger regions.

The procedure began by determining the seed of each group image. This was identified using the "largest connected component" method to calculate all connected regions in each image. The largest one image was then selected as the initial seed of the image.

After defining the seed regions, the mean grayscale values of T1 and T2 images of the seed region were calculated. The normal distribution statistical concept was applied to determine the merging criteria: for each pixel adjoining the seed region, if the T1 and T2 grayscale lies within two standard deviations (SD) from the mean grayscale of the seed region, this pixel is merged into the seed region. If no more neighboring pixels exist with a grayscale level within 2SD, the merging process stops.

**Figure 4:**
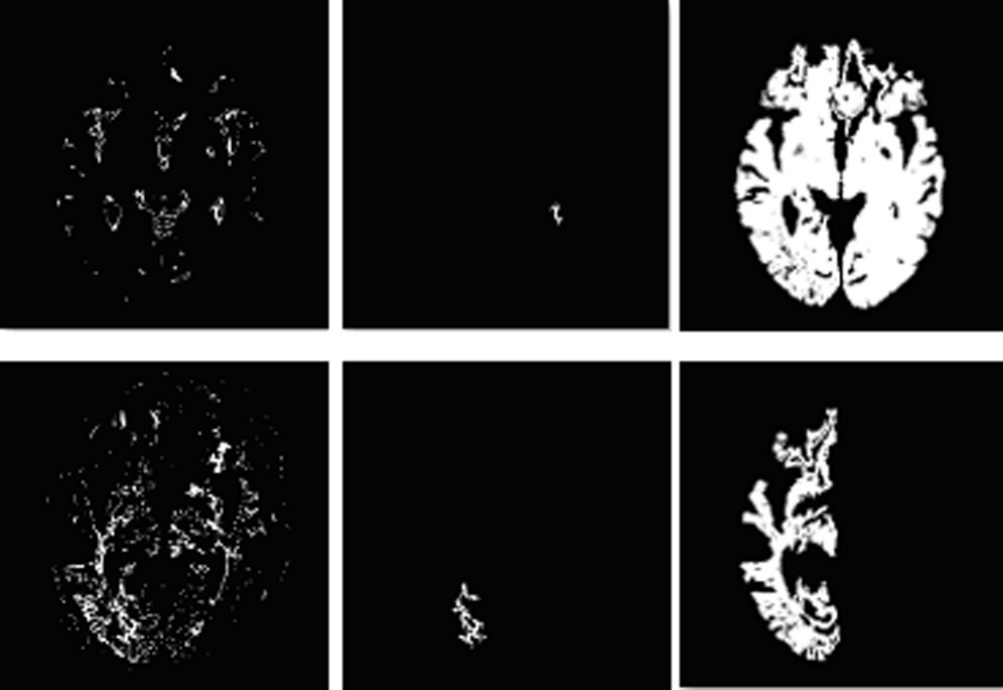
**Result after Region growing**. After FCM clustering, some image may be too fragmenting to be properly classified (upper left and lower left). In this occasion, a seed area is selected within the image (upper middle and lower middle), and after pixel aggregation, these fragment could grow into more meaningful image. The upper row image now could be identified as tumor-containing image, where as the lower row image will be classified as background, and be readily processed at later stage.

Using this method, the original fragmented image started to become more meaningful, whether it was with or without favorable features, as shown in Figure 4. The presence or otherwise of favorable features, could then be used as criteria for keeping the image, as tumor-containing, or eliminating it, as background, at the subsequent knowledge-based stage..

### Knowledge-based techniques

In this step expert knowledge was used to extract the brain tumor from the 32 results existing after the region-growing stage. Four types of knowledge were applied to select tumor image groups and exclude non-tumor groups:

(a) Screen the image according to size: the bounding box method

**Figure 5:**
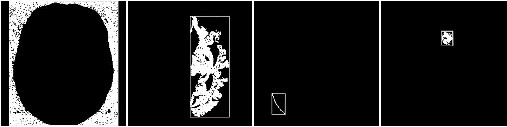
**The bounding box method**. Using brain tissue image (left 1) as standard, the image group whose image box is larger than 1/2 height and width of the brain image will be excluded (left 2), otherwise will be preserved (right 1 and 2).

For each set image the height and width of a box surrounding the whole image containing tissue was calculated. Considering that the size of the tumor is seldom more than half the size of the brain tissue, any image group with an image box larger than half the height and width of the brain image could be excluded, as shown in Figure 5.

(b) Decide which side of the brain the tumor is on: modified symmetrical-histogram analysis

Traditional symmetrical-histogram analysis can detect tumors located in either brain hemisphere, but fails to locate tumors located at the midline brain [31]. In this research, a correlation coefficient was applied to overcome this problem. Using the knowledge that tumors have a brighter grayscale value on T2-weighted images, and with the precondition that all brain images were well-sliced and well-centered, the histograms of the right and left side brain were generated. Pixels with grayscale values of below 50 were then removed as noise. A correlation coefficient was applied to compare the difference between the left and right side histograms. A correlation coefficient < 0.95 denotes that the differences between both histograms are statistically correlated, and the tumor is situated on either side of the hemisphere. In contrast, a correlation coefficient > 0.95 denotes no statistical difference between both side histograms, meaning that the tumor might be located in the middle of the brain hemisphere.

**Figure 6:**
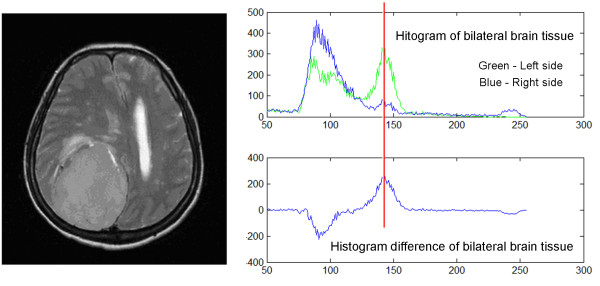
**The difference of bilateral side histogram analysis**. By analyzing the T2-weighted MR image (left), we obtained the histogram of bilateral brain tissue (right upper) and the curve represented their difference (right lower). Using the highest absolute value in histogram difference curve as reference point (red line), here the grayscale value of both sides histogram were compared, and the side with higher grayscale value is likely to have tumor. In this case, the left (green) side had higher grayscale at reference point, so the tumor is on the left side of the brain.

Using the above procedure, image groups with correlation coefficients < 0.95, went through further analysis to determine on which side the tumor was located. This was performed using the absolute value in the histogram difference as a reference point, and comparing the grayscale values of both sides to this point. As shown in Figure 6, the side with the higher grayscale value is likely to contain the tumor. The image group containing tissue from the other side of the brain was therefore eliminated.

(c) Define the brain tumor image based on the solidity

The definition of solidity in this context can be expressed as follows:

*Solidity = image area/the smallest convex polygon that could cover the image*.

**Figure 7:**
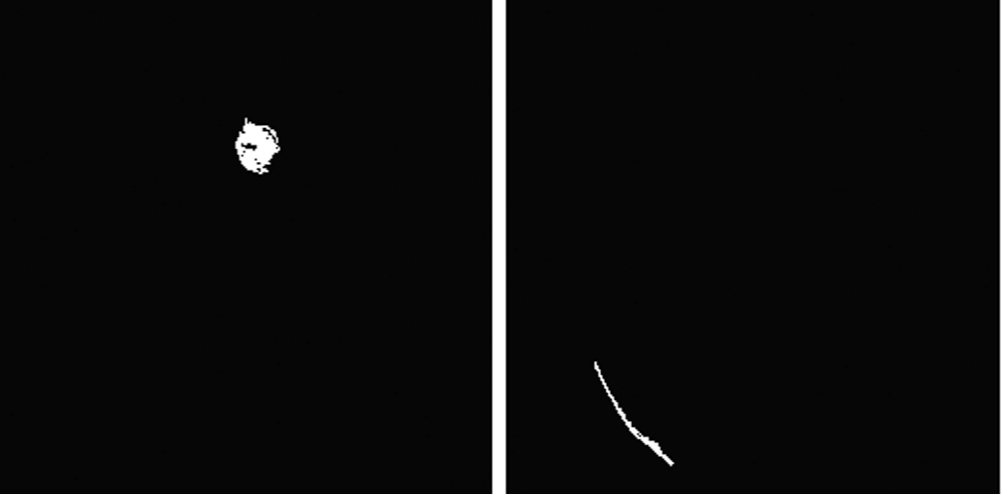
**The solidity of tumor image**. *Left: *image with high solidity (78.99) more likely to be tumor; *Right: *image with low solidity (39.77) more likely to be normal tissue.

Usually, a tumor has a higher solidity value than normal brain tissue, as shown in Figure 7. This research, therefore, used the agglomerative hierarchical clustering algorithm [32], rather than traditional definite value criteria to remove the groups with low solidity value. The procedure can be summarized as follows:

*Step1*. Assuming n data needs to be separated into c groups, first define every data set as a cluster c_i_, i = 1,..., n.

*Step2*. Calculate the distance between these clusters. Clusters with the nearest distance will be the closest clusters: c_i _and c_j_.

*Step3*. Merge c_i _and c_j _to new cluster.

*Step4*. If the number of clusters has reached 1, or the desired number, then stop; otherwise repeat steps 2 and 3.

This "bottom-up" clustering method allowed the separation of data into groups with higher and lower solidity. Groups with tumor tissue were included in the first group, and those in the second group were eliminated.

(d) Eliminate the non-tumor area based on the tissue area

After completing the previous screening procedure, the remaining groups included images with small area noise, or images with a larger area, which could be tumor, edema tissue or other meaningful tissues. Agglomerative hierarchical clustering was then applied again, except this time the "area" parameter was screened. Groups with little tissue area were eliminated.

At this point an alternative semi-supervised procedure was also employed in order to further validate results, and for comparison with the original automatic pathway. In this procedure the tumor-containing image was selected manually, and the results were compared with those from the automatic pathway, and with the ground truth.

Finally, if results contained more than one image group, they were put through the judgment of the logical "or" and merged into one image. The preliminary brain tumor image defined by the system was thus produced.

### Morphological image processing

After the region-growing procedure, there are many residual areas around the tumor region. Also, if the tumor is not homogenous, there will tend to be small holes within the tumor mass. The morphological procedure is therefore used on these binary images to refine the margin and content of the tumor images.

Firstly, the erosion operator was employed to separate the residual neighbor area from the tumor, and then the holes within the tumor mass were filled by dilation. The resulting tumor mass was smoother at the margin and more solid in content. This completed the process of brain tumor segmentation.

### Validation of segmentation results

Results from the present study's image segmentation system (SYS) were compared with the ground truth (GT); images segmented manually by medical experts. In this study, all images, including the contrast-enhanced ones, were reviewed independently by two board-certified neurosurgeons (CL and FX). These two experts contoured the tumors based on the contrast-enhanced T1-weighted MR images (CET1), using the same contour method for the planning of radiosurgery of brain tumors. If some challenging condition was encountered, such as the presence of "dural tails" (a common phenomenon existing in the contrasted MR images of meningiomas), this would therefore be adequately covered by the experts. Contours made by CL were arbitrarily chosen as the GT, and the reproducibility of the two observers was calculated.

To quantify the differences between the SYS and the GT at pixel level, results were classified into the following:

• *True positives *(TP), where the SYS and the GT both classified an image as a tumor.

• *True negatives *(TN), where the SYS and the GT both classified an image as normal tissue.

• *False positives *(FP), where the SYS classified an image as tumor but the GT as normal tissue.

• *False negatives *(FN), where the GT classified an image as tumor but the SYS as normal tissue.

To demonstrate the accuracy and efficiency of the system as objectively as possible, two well-established and commonly used scoring parameters: the *percent match *(PM) and *correspondence ratio *(CR) [18,20,21], were applied to scrutinize the results with the definitions: *PM = TP/GT, CR = (TP - (0.5 × FP))/GT*. The PM value uses the manually generated ground truth as a standard to calculate the accuracy of the system. If the PM value is 100%, all SYS tumor images segmented by the system also exist in the ground truth, and vice versa. Concerning CR, the closer the value is to 1, the closer the system judgment is to the ground truth. Thus, if a system has a PM value close to 100%, but the CR value is negative, then this system is judged to have good accuracy but high error, so such a system is not ideal.

Using the above parameters, the PM and CR of the automatic method, and also the semi-supervised alternative, were calculated. Statistical methods were employed to verify the significances of differences. Using the same methods, the entire automatic pathway was repeated, this time without incorporating the region-growing process. The results were then compared with those from the original automatic pathway.

## Results

**Figure 8:**
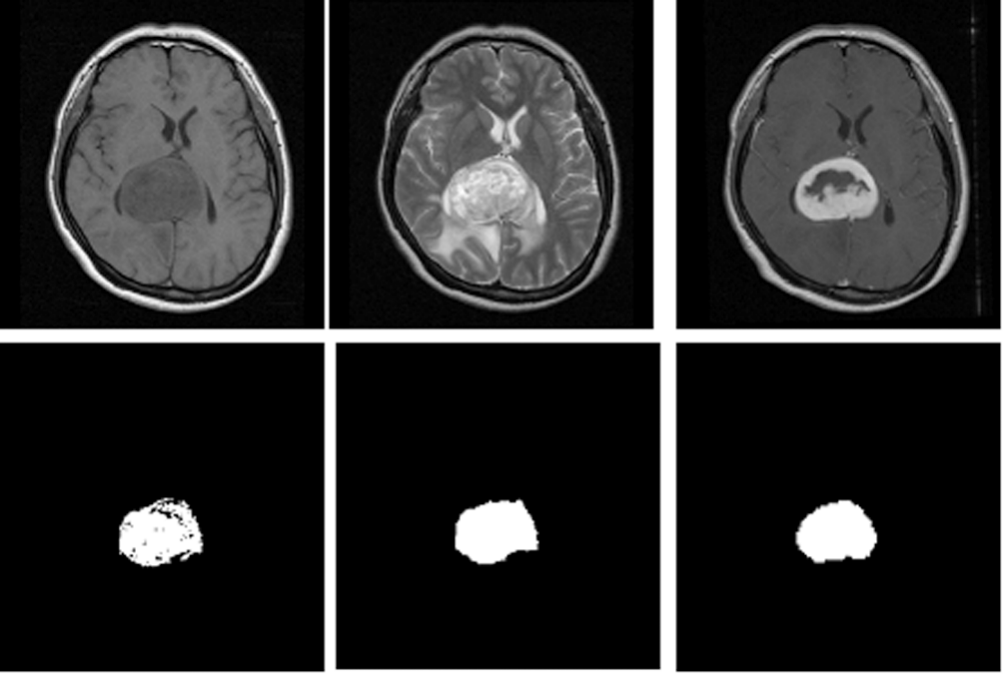
**The result of tumor segmentation**. One of the result were shown here, the original non-contrasted T1 (upper left) and T2 -weighted (upper middle) MR image were processed. The tumor image segmented by semi-supervised method (lower left) and automatic method (lower middle) were compared with "ground truth" (lower right), which was manually segmented from contrasted-enhanced T1 image (upper right).

**Figure 9:**
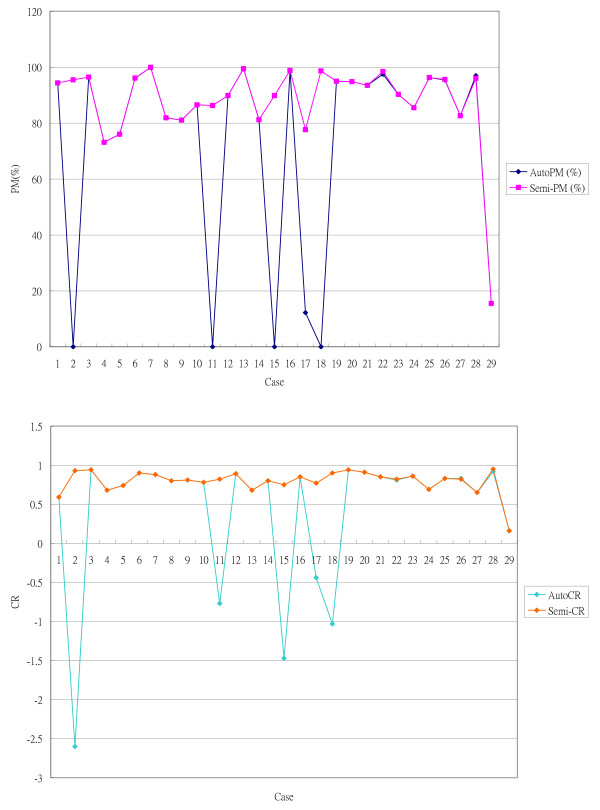
**Diagram showing quantified evaluation of the results obtained by automatic pathway and semi-supervised alternative**. *Upper: *percent match (PM) curves of 29 cases; *Lower: *correspondence ratio (CR) curves of 29 cases in two groups. We can see in case no. 2, 11, 15, 17 and 18, low PM and CR were observed in automate pathway but not in semi-supervised group; Case 29 show poor PM but good CR in both groups. Beside these cases, some trace difference could be observed in case no. 22, 26 and 28.

Of 29 groups of non-contrasted T1 and T2-weighted MR images of meningioma, the automatic algorithm successfully segmented 23 groups, one example of which is shown in Figure 8. The overall PM value was 72.80 ± 36.20% and the CR value was 0.43 ± 0.86. Table 1 presents the detailed result values of this automatic system. Of the 6 failed cases, one (case no. 29) was also the only failure case in the semi-supervised group. The semi-supervised alternative (in which the tumor image was picked manually instead of going through the agglomerative hierarchical clustering at the last stage of KB procedure) successfully segmented 28 out of the 29 groups. The PM value was 87.82 ± 15.91%, and the CR value was 0.79 ± 0.15. This method was, therefore, better than the automatic pathway. The paired t-test confirmed results, providing a P value of 0.02344 (< 0.05) for PM, and 0.03392 (< 0.05) for CR; significant differences in findings. Overall results are presented as a diagram in Figure 9.

**Table 1:** Results obtained by the automatic system

Case	GT	SYS	TP	FP	FN	TN	PM (%)	CR
1	2132	3512	2012	1500	120	61904	94.37	0.59
2	855	4440	0	4440	855	60241	0.00	-2.60
3	4271	4313	4118	195	153	61070	96.42	0.94
4	1183	990	865	125	318	64228	73.12	0.68
5	4517	3577	3434	143	1083	60876	76.02	0.74
6	2270	2452	2180	272	90	62994	96.04	0.90
7	1100	1370	1099	271	1	64165	99.91	0.88
8	761	648	623	25	138	64750	81.87	0.80
9	5607	4559	4544	15	1063	59914	81.04	0.81
10	1081	1126	935	191	146	64264	86.49	0.78
11	2013	3104	0	3104	2013	60419	0.00	-0.77
12	2500	2298	2246	52	254	62984	89.84	0.89
13	1968	3198	1957	1241	11	62327	99.44	0.68
14	442	374	359	15	83	65079	81.22	0.80
15	942	2764	0	2764	942	61830	0.00	-1.47
16	5549	6967	5482	1485	67	58502	98.79	0.85
17	2137	2657	261	2396	1876	61003	12.21	-0.44
18	732	1508	0	1508	732	63296	0.00	-1.03
19	4379	4205	4158	47	221	61110	94.95	0.94
20	2589	2650	2455	195	134	62752	94.82	0.91
21	1397	1554	1306	248	91	63891	93.49	0.85
22	4702	6114	4584	1530	118	59304	97.49	0.81
23	1560	1537	1408	129	152	63847	90.26	0.86
24	1384	1650	1183	467	201	63685	85.48	0.69
25	588	719	566	153	22	64795	96.26	0.83
26	1662	2012	1586	426	76	245750	95.43	0.83
27	1715	2012	1417	595	298	245530	82.62	0.65
28	4495	4825	4359	466	136	242870	96.97	0.92
29	1329	206	206	0	1123	246510	15.50	0.16

The results were demonstrated at pixel level, together with their validation parameters. *GT: *Ground Truth*; SYS: *Automatic System*; TP: *True Positive*; FP: *False Positive*; FN: *False Negative*; TN: *True Negative*; PM: *Percent Match*; CR: *Correspondence Ratio*.

### Concordance of manual segmentation

Testing of the reproducibility of the manual method using the contours made by CL as the GT provided a mean PM value, from FX's contouring, of 99.41 ± 1.44% and a mean CR of 0.89 ± 0.06. Reversing the testing criteria, using FX's contouring as the GT, provided a mean PM, from CL's contouring, of 83.58 ± 7.77% and a mean CR of 0.83 ± 0.07. The contours made by FX tended to be more generous (p < 0.001, paired t-test).

### Comparing the results with and without region growing

Applying FCM clustering without incorporating the region-growing method, produced significantly worse results. For 9 cases PM = 0 and overall PM = 25.32, CR = 0.23. These results are significantly less accurate than the results from FCM incorporating the region growing algorithm.

### The results of midline tumor detection

Of 29 cases, 6 cases with tumors situated at the midline, as confirmed by the GT, were all successfully detected, both by the automatic method and by the semi-supervised alternative.

## Discussion

By using only 2 non-contrasted MR images, the overall results of our automatic method for segmentation of the meningioma were comparable to those from other research [18,20,21]. Adding the region-growing algorithm proved to be a crucial element in the study Without this procedure, the PM and CR values were significantly lower, even in the semi-supervised alternative. Here the noisy result after FCM clustering tended to cause more error in the subsequent KB selection process, and causes a significant increase in the calculation load. The region-growing algorithm incorporated the neighbor factor into the clustering and this resulting in more meaningful data, greater accuracy in subsequent analysis, and an increased calculation efficiency.

In the knowledge-based selection algorithm, a relative value, rather than an absolute value, was applied. This approach meant that no training data was necessary. If noticeable edema tissue existed within the image, however, this would cause error in these KB procedures because, like tumor tissue, these tissues have a high grayscale value in T2-weighted images. This is the main reason for the six failure cases using the automatic method. Inspection of these T2-weighted MR images identified large areas of edema tissue in 5 cases. At this point, use of a semi-supervised method would have overcome the deficit. This is the reason for more accurate overall PM and CR values using the semi-supervised alternative method than the automatic pathway. Excepting the 5 failed cases, however, the automatic pathway showed good accuracy and satisfactory error rates; (PM = 87.41 ± 17.16% versus 87.46 ± 17.19%; CR = 0.78 ± 0.16 versus 0.78 ± 0.16) and comparable findings to those from the semi-supervised group. The knowledge-based selection applied in the present study, therefore, provides relatively stable and reliable conditions compared to the absolute value screening. Results also imply that, if patient selection excluded the edema cases, the overall results for the automatic pathway would be greatly improved. In the future, in order to effectively resolve the edema tissue problem, more MR sequences, such as Fluid Attenuated Inversion Recovery (FLAIR) images, could be included as materials. Newer MR imaging techniques, such as Dynamic Susceptibility Contrast (DSC) and Dynamic Contrast Enhanced (DCE) imaging [20], could also be incorporated into future work.

Case no. 29 was the unusual case as it failed in both automatic and semi-supervised groups (Figure 9). In the other 5 cases which failed in the automatic pathway, both the PM and CR values were significantly inferior to the semi-supervised alternative. In this case, however, the PM and CR values were the same in both groups, with low PM, but good CR, values. This indicated a poor detection rate but good correspondence. Inspection of the MR images revealed the absence of prominent edema tissue, which may account for the good CR value, as false grouping of normal tissue into the tumor image would be minimal. The heterogeneous character of the tumor image, which results in partial detection of the tumor tissue, may account for the low PM value.

**Figure 10:**
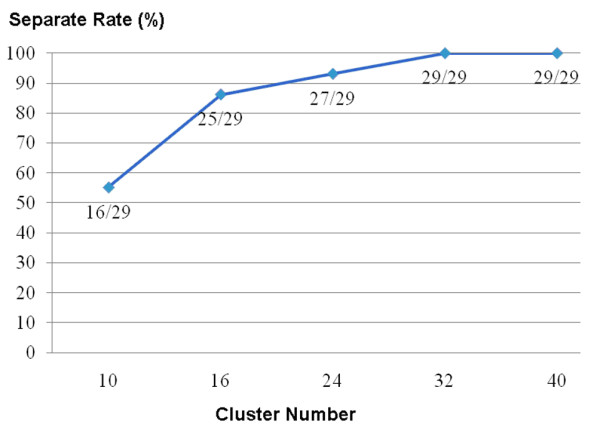
**The relation between cluster number in FCM and tumor image separation rate**. Could see the separation rate is improving as cluster number increase; finally come to a plateau when cluster numbers exceed the number of 32.

During the research design phase, one of the most challenging tasks was determining the best cluster number for analysis. A previous study proposed a cluster number of 10 [21], but, in the present research, 10 clusters were not enough to aggregate the tumor images in every group. Since the brain is mostly composed of eight kinds of tissue, each with distinct grayscales (gray matter, white matter, CSF, skull, air, tumor, edema and normal tissue), multiples of eight cluster numbers were assayed: 10, 16, 24, 32 and 40. After clustering with fuzzy c-mean, the separation rate of tumor stand alone positively correlated with the cluster number, and reached a maximum after 32, as shown in Figure 10. However, the calculation load also increased with the cluster number, therefore the number of 32 was selected as the cluster number of choice.

The second major goal of this study was to effectively detect menigioma located at the midline position of the brain. The automatic pathway successfully detected 100% of the midline tumor cases by improving on the traditional approach for tumor location and incorporating the concept of correlation. This stage was important as it avoided potential failure caused by midline tumors, which may have occurred in other related works, and contributed to overall good results.

The final section of the study was to examine the reproducibility of the methods. The PM and CR values indicated differences between the two human operators. In this respect, an automatic method is superior to a manual method as machines will usually generate the same results using the same materials and methods. Based on their unique character, the fuzzy-based algorithm tended to provide slightly different results every time the calculation was repeated, but the differences were so trivial that they could be ignored. Figure 9 reveals the slight differences in PM and CR values for the automatic pathway compared with the semi-supervised alternatives in three cases. Manual elimination of some of the image groups during the final stage of the KB selection in the semi-supervised method, but not in the automatic pathway may have caused these variations. These slight differences did not influence the overall results.

## Conclusions

This research demonstrates the possibility of using only two non-contrast enhanced MR images -T1 and T2 images - for brain tumor segmentation. The algorithm integrates the fuzzy-c-mean and region growing techniques and successfully detects meningiomas, even in the brain midline. Compared with the ground truth, this quantifiable method shows a feasible detection rate and good accordance. The system, therefore, demonstrates high potential for practical clinical use, and may assist medical experts with tumor location, volumetric estimation, therapeutic planning and follow-up. Future aims include the improvement of the system's calculation loading, exploring the potential use of other MR modalities, and experimenting with the 3D image format.

## Competing interests

The authors declare that they have no competing interests.

## Authors' contributions

TMH participated in the design of the study, interpretation of the data, and involved in drafting and revising of the manuscript. YL performed the FCM process and statistical analysis, participated in the drafting of the manuscript. CL conceived the study and participated in its design, also contribute to data acquisition. FX also conceived of the study, and participated in its design and data analysis. IC participated in analysis and interpretation of the data and coordinated the drafting of the manuscript. JW was contributed to the analysis and interpretation of the data and given final approval of the version to be published. All authors read and approved the final manuscript.

## Pre-publication history

The pre-publication history for this paper can be accessed here:

http://www.biomedcentral.com/1472-6947/11/54/prepub
